# Liver Subcapsular Hematoma Conservative Treatment in HELLP (Hemolysis, Elevated Liver Enzymes, and Low Platelet Count) Syndrome: A Case Report

**DOI:** 10.7759/cureus.96005

**Published:** 2025-11-03

**Authors:** Elizabeth Aide Gómez Vega, Edgar Mendoza Reyes, Abril A Arellano Llamas, Flor María G Arroyo Cano, Zarela L Chinolla Arellano

**Affiliations:** 1 Obstetrics and Gynecology, Unidad Médica de Alta Especialidad, Hospital de Ginecología y Obstetricia No.3 "Dr Víctor Manual Espinosa De los Reyes Sánchez" Centro Médico Nacional La Raza, México, MEX; 2 Pediatric Endocrinology, Unidad Médica de Alta Especialidad, Hospital de Ginecología y Obstetricia No.3 "Dr Víctor Manual Espinosa De los Reyes Sánchez" Centro Médico Nacional La Raza, México, MEX; 3 Intensive Care Unit, Unidad Médica de Alta Especialidad, Hospital de Ginecología y Obstetricia No.3 "Dr Víctor Manual Espinosa De los Reyes Sánchez" Centro Médico Nacional La Raza, México, MEX

**Keywords:** cesarean section, conservative management hepatic hematoma, hellp syndrome complications, hepatic bleeding, hepatic subcapsular hematoma, preeclampsia

## Abstract

Subcapsular hepatic hematoma is a life-threatening complication during pregnancy. Conservative treatment could be possible with an equipped unit and a fully staffed team available 24 hours a day. We present the case of a 40-year-old patient at 37 weeks of gestation with preeclampsia meeting severe features who presented with diffuse abdominal pain radiating to the right shoulder. In a private hospital, during a cesarean section, hepatic bleeding was observed. In our hospital, partial HELLP (Hemolysis, Elevated liver enzymes, and Low Platelet count) syndrome was diagnosed, and treatment began. Once stabilized, a CT scan revealed two subcapsular hepatic hematomas with a total volume of 1700 cc. The multidisciplinary team adopted a conservative management approach, incorporating close clinical and biochemical follow-up. After 25 days, the patient was discharged home with a significant reduction in the size of both hematomas.

## Introduction

Hypertensive diseases affect 5-10% of pregnancies [1]. Preeclampsia, a high blood pressure complication, is the second cause of maternal death in Mexico [2]. HELLP (Hemolysis, Elevated Liver Enzymes, and Low Platelet Count) syndrome is characterized by hemolysis (lactate dehydrogenase >600UI/L), elevated liver enzymes and decreased platelet count to <100,000 platelets/μL [3,4]. It affects 0.5-0.9% of pregnancies and has a mortality greater than 25% [5].

Subcapsular hepatic hematoma (SHH) affects 0.9-1.6% of patients with HELLP syndrome, meaning an approximate 1/250,000 pregnancies [6]. Maternal case fatality rate varies from 18% to 86% and perinatal mortality can reach 80% [4]. SHH is explained by a microangiopathic syndrome with hemolysis, which starts with endothelial dysfunction and intravascular fibrin deposition leading to obstruction of the hepatic sinusoidal spaces, causing intrahepatic vascular congestion, distension of Glisson's capsule, and formation of a subcapsular hematoma located most frequently in the anterosuperior region of the right hepatic lobe [7]. It is believed that the imbalance between the pro- and anti-angiogenic factors leads to endothelial dysfunction, vasospasm, reduced blood flow, liver infarction, microvascular progressive bleed, and hematoma formation [6]. Histopathology has revealed periportal hemorrhage, microvesicular lipid infiltration in the hepatocytes, leading to necrosis [6]; these findings correlate with the clinical case severity.

The characteristic symptoms are pain in the right upper quadrant derived from Glisson capsule distention, general malaise in 90% of cases, nausea and vomiting in 50% [3], evidence of hypovolemic shock due to blood sequestration within a developing hematoma or its rupture, leading to a potentially catastrophic outcome [8].

In pregnancy, shock symptoms could be wrongly interpreted as thromboembolism, pancreatitis, or other hepatobiliary syndromes. The diagnostic time lapse is crucial for the patient to get the correct treatment and be taken to a facility with complete equipment and staff to attend this critical diagnosis. This report highlights clinical decision-making and non-operative care criteria for the successful conservative management of a rare, huge bilateral subcapsular hepatic hematoma in a patient with HELLP syndrome.

## Case presentation

A 40-year-old woman with two previous pregnancies, no comorbidities, a normal pregnancy of 36 weeks, and adequate prenatal care presented to the emergency department of a private hospital with general malaise, hypogastrium pain radiating to the shoulder and right scapular region (Day 0). Systolic blood pressure was above 140 mmHg, but this unit did not register the specific pressure; she did not receive any treatment and was sent home.

Two days later (Day 2), she returned to that hospital because her symptoms worsened. Her blood pressure increased to 170/90 mmHg, indicating preeclampsia with severity criteria, and pregnancy was ended by cesarean. During surgery, there was blood in the abdominal cavity, approximately 150 mL, apparently coming from the hepatic region; a Penrose draining system was placed. She delivered a live newborn weighing 2645 grams, and the APGAR grade was 9/9. There was no additional perinatal history, and the newborn was healthy.

While at the same hospital, the next day (Day 3), transaminases of the patient were elevated, and an ultrasound showed a hypoechoic image in the left lobe measuring 46 x 36 x 40 mm, volume 34 cc, and was considered a possible hepatic hematoma. She was sent to our medical unit by ambulance. Upon arrival, she presented stable vital signs, blood pressure 138/81 mmHg, heart rate 78 beats per minute, respiratory rate 14 breaths per minute, temperature 36.5°, and oxygen saturation 95%. Clinically, she reported persistent right upper quadrant pain. Based on the biochemical context shown in Table 1, she was diagnosed with post-surgical puerperium, preeclampsia with severe features, HELLP syndrome, and suspected hepatic hematoma rupture.

**Table 1:** Biochemical parameters from initial presentation through follow-up Serial biochemical parameters show progressive improvement in hepatic function, platelet count, and LDH levels during conservative management

Parameters	Reference Range	Patient values, day 2		Patient values, day 3	Patient values, day 6	Patient values, day 10	Patient values, day 14	Patient values, day 20	Patient values, day 27
Glucose (mg/dL)	70 -105	129		76.8	62.1	74.6	147	101	92.7
Urea (mg/dL)	15 -38	45		-	29.6	35.5	29.6	20.1	26.4
Creatinine (mg/dL)	0.6 - 1.1	0.85		0.97	0.42	0.5	0.59	0.44	0.56
Uric Acid (mg/dL)	2.6-6.0	8.3		6.26	4.76	3.42	3.23	2.67	3.65
Amino Transferase (UI/L)	13 - 40	426		486	50.6	60.8	42.8	41.6	27.5
Alanine Transferase (UI/L)	40 -150	296		311	90.8	67.1	32.1	32.6	20
Alkaline Phosphatase (UI/L)	125-220	200		161	108	146	131	129	141
Lactate Dehydrogenase (UI/L)	125-220	1020		750	448	860	856	797	583
Leukocytes (K/mcl)	4.8-10.8	11.1		14.7	11.0	10.3	17	8.3	
Hemoglobin(g/dL)	12-16	11.9		9.4	8.4	9.9	9.8	9.3	
Hematocrit(%)	37-47	28.8		27.1	24.6	29.8	29.6	28.8	
Platelets (K/uL)	150-450	143		123	191	384	491	484	
Thrombin Time (seconds)	9.0-13	13.3		10.1	-	11.8	12.0		
Prothrombin Time (seconds)	25.3-37.5	32.5		33.1	-	33.2	36.0		
International Normalized Ratio (%)	0.9-1.5	1.04		0.9	-	1.0	1.0		
Urinary pH	4.6-8.0	-		5.0					
Specific Urinary Gravity	1.003-1.035	-		1.005					
Urinary Leukocytes	Negative	-		Negative					
Urinary Nitrites	Negative	-		Negative					
Urinary Proteins	Negative	-		Negative					
Urinary Hemoglobin	Negative	-		Negative					

**Figure 1:**
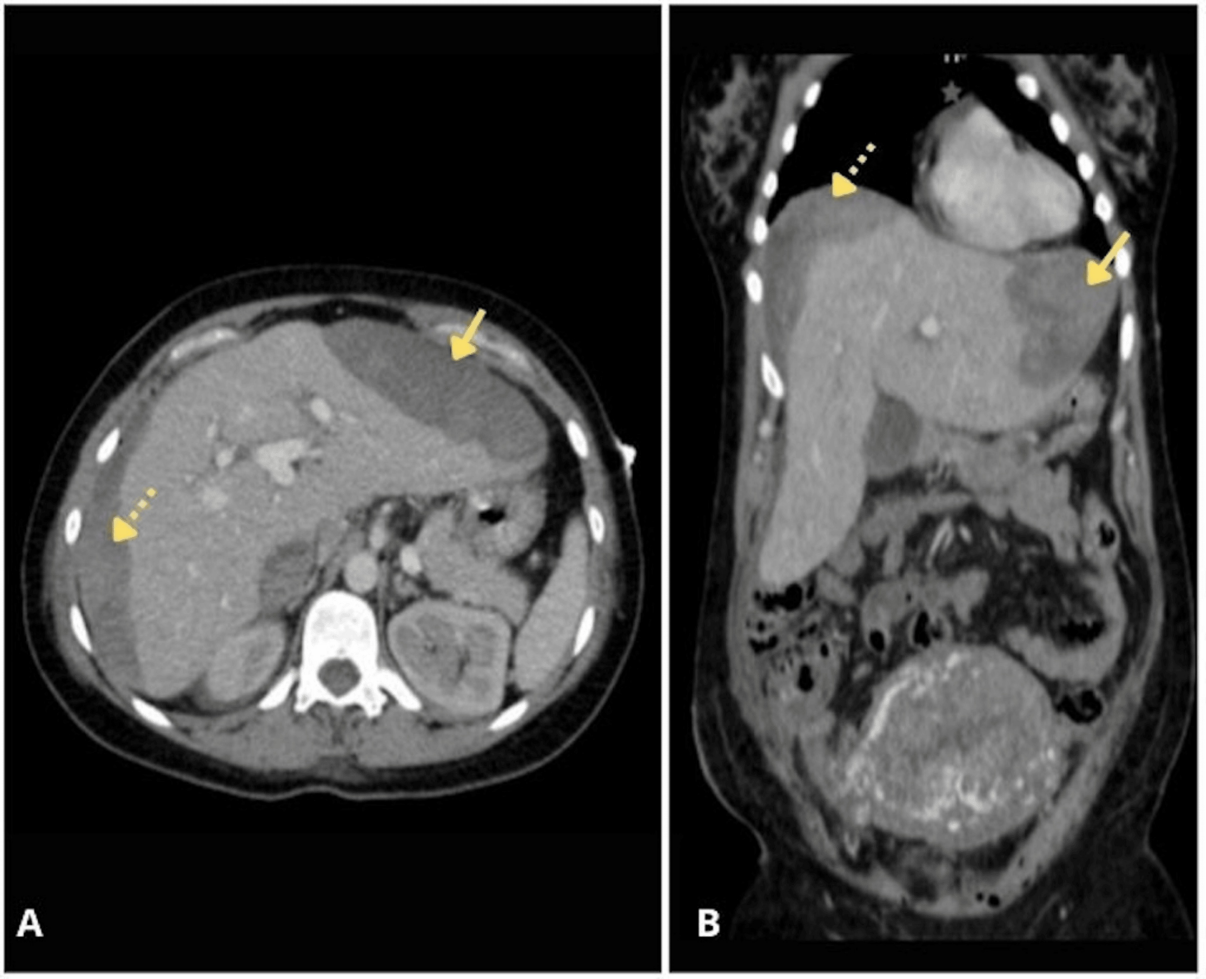
Baseline CT imaging (contrast-enhanced). A: Axial view showing large right-lobe subcapsular hematoma (dotted arrow) and smaller left-lobe lesion (solid arrow). B: Coronal reconstruction demonstrating bilateral involvement, total estimated volume ≈1,790 cc.

The patient was directly admitted to the intensive care unit (ICU), the same day of her referral. Antihypertensive treatment was initiated with a calcium channel blocker (nifedipine 30 mg orally every eight hours) and a beta-blocker (metoprolol 100 mg orally every 12 hours), along with magnesium sulfate for neuroprotection at 1 g/hour for 24 hours, and a CT abdominal scan uppon admission revealed subcapsular hematoma measuring 191 × 140 × 107 mm (volume: 1496 cc) in the right hepatic lobe, and another lesion in the left lobe measuring 103 × 46 × 119 mm (volume: 294 cc), for a total estimated volume of 1790 cc (Figure 1).

Given the patient’s hemodynamic stability, the medical team opted to maintain clinical observation. Due to the patient’s delicate condition, a follow-up ultrasound was performed on the fifth day, revealing a lesion in the left hepatic lobe measuring 46 × 66 × 92 mm (volume: 148 cc) and a fluid collection in the right hepatic lobe measuring 127 × 50 × 119 mm (volume: 398 cc).

**Figure 2:**
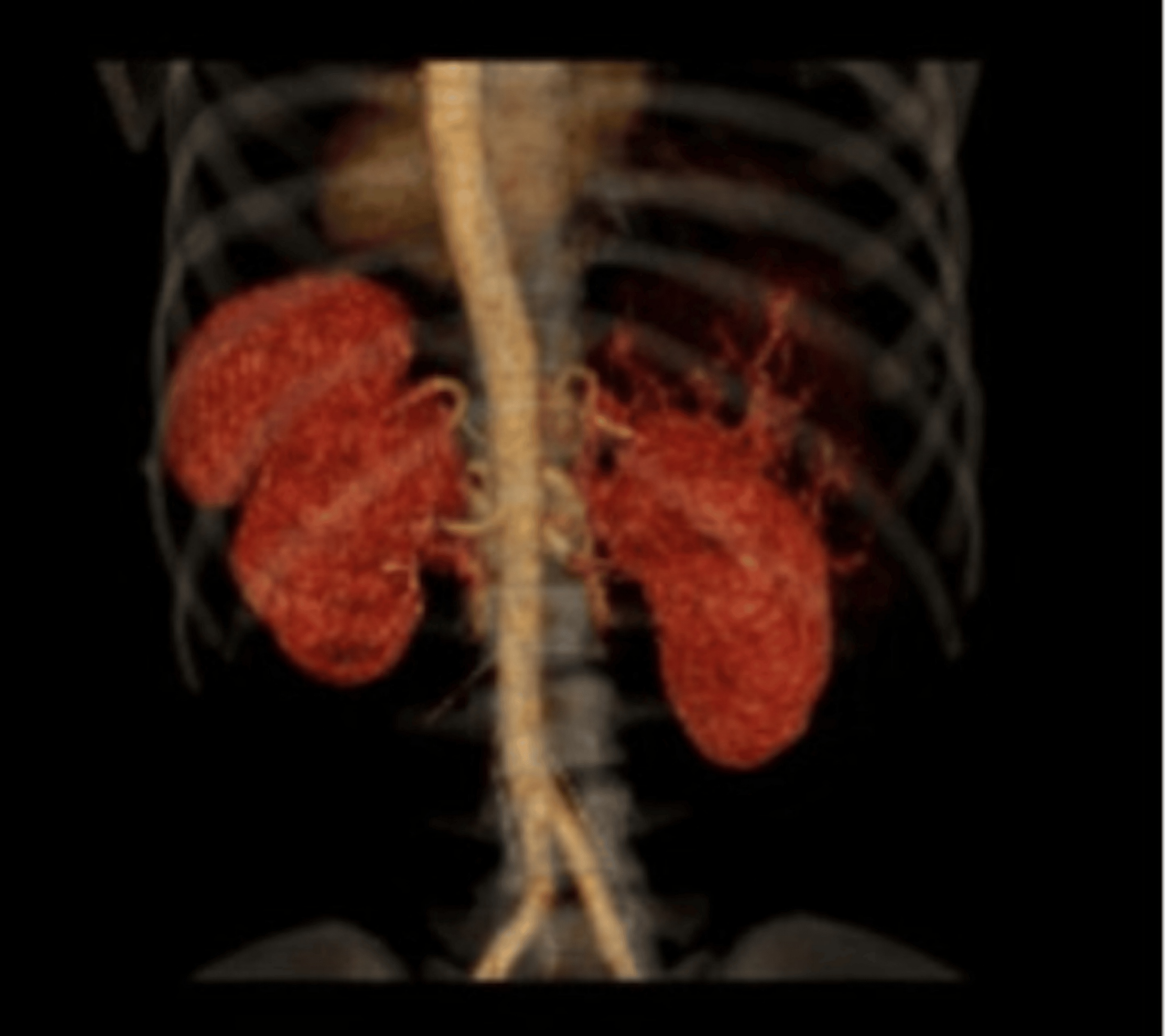
Three-dimensional reconstruction of the liver performed one week after admission Showing bilateral subcapsular hematomas (right lobe ≈1550 cc, left lobe ≈131 cc). Visualization generated with a Philips Brilliance 64 CT scanner (Koninklijke Philips N.V., Amsterdam, Netherlands). No new lesions or signs of active bleeding were observed.

Hepatic arteriography was performed on Day 11, to rule out active bleeding or arterial extravasation, which would have required immediate radiologic or surgical intervention. In this case, arteriography confirmed the absence of active bleeding, supporting the decision to continue with conservative management, and provided detailed information on liver vascularization that facilitated planning of clinical and imaging follow-up (Figure 2).

The multidisciplinary team's opinion was divided; the general surgery team recommended surgical intervention due to the size of the hematoma in the right lobe, but the Gynecology and Obstetrics service proposed conservative management, as there was no evidence of hepatocellular injury, capsular rupture, active bleeding, or hemodynamic instability. In consensus with the intensive care unit, the team decided on conservative treatment. 

The patient remained in close hemodynamic clinical, laboratory, and imaging surveillance in the intensive care unit (ICU) for 19 days. She was treated nil per os (nothing by mouth) for three days, received parenteral analgesia, and her hypertension was controlled. Table 2 summarizes the progression of the imaging findings. The hemodynamic stability was assessed every hour in the intensive care unit; there was no instability in her observation period. The parameters used for this aim were: tissue perfusion, systolic blood pressure <90 mmHg or a drop >40 mmHg from baseline, sustained tachycardia, oliguria, altered consciousness, pallor, profuse sweating, and evidence of peripheral hypoperfusion. However, she required a red blood cell concentrate with the lowest hemoglobin level, which was 8.4 g/dL.

**Table 2:** Follow-up of subcapsular hematoma volume

Event (day)	Right Lobe Hematoma Dimensions (mm)	Right Lobe Hematoma Volume (mL)	Left Lobe Hematoma Dimensions (mm)	Left Lobe Hematoma Volume (mL)	Others
Ultrasounds					
Day 2	Not applicable	Not applicable	46x36x40	34	NA
Day 5	127x50x119	398	46x66x92	148	NA
Day 9	100x75x90	358	35x34x75	114	Hepatorenal space collection with a volume of 10 mL
Day 37	83x36x65	102	45x34x44	35	NA
Day 68	42x23x55 24x8x13	25 1.3	30x20x28	9	NA
CT scans					
Day 3	191x140x107	1496	103x46x119	294	NA
Day 11	184x179x90	1550	81x29x107	131	NA
Day 19	195x79x133	1000	110x73x37	148	NA
Day 31	160x108x68	614	97.8x30x103	158	NA

With appropriate management, blood pressure was controlled by the fifth day of hospitalization, allowing discontinuation of the beta-blocker. The calcium channel blocker was withdrawn on the sixth day, and the patient was discharged without antihypertensive therapy.

The patient began ambulation, and we removed the Penrose drain on Day 13. During her stay in the ICU, the patient was assessed by general surgery, gastroenterology, nutrition, and psychology. Imaging follow-up revealed, on Day 19, that the hematoma volume had begun to shrink, and given her clinical improvement, surveillance continued on the Perinatology ward on Day 20, where mechanical and pharmacological thromboprophylaxis began.

**Figure 3:**
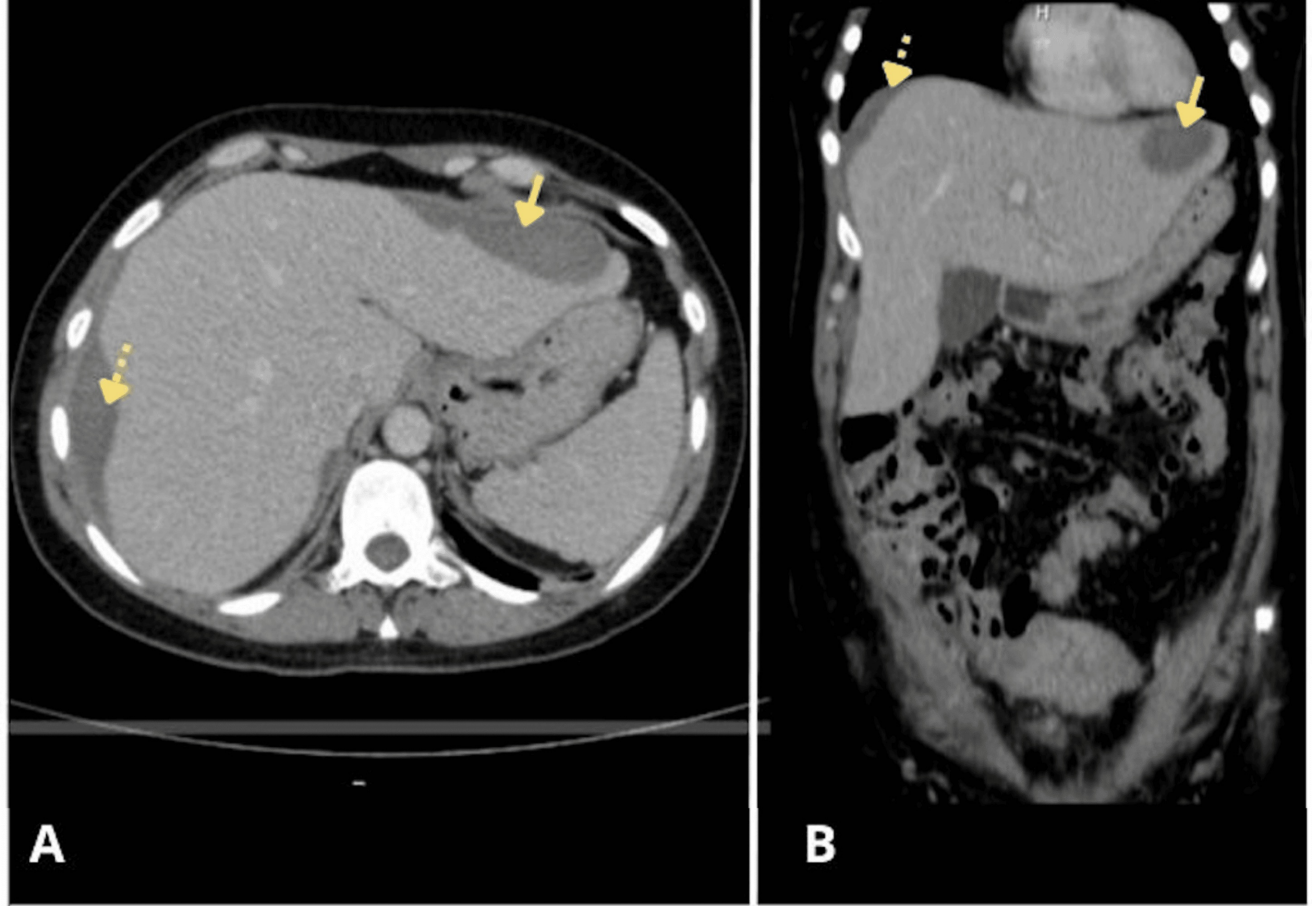
Follow-up CT at 28 days post admission. A: Axial view showing marked reduction of right-lobe hematoma (≈614 cc) and left-lobe hematoma (≈158 cc). B: Coronal view demonstrating preserved liver architecture, no capsular rupture, and progressive resolution.

After 27 days of hospitalization, the medical team discharged the patient home. A follow-up CT scan performed four days later showed a marked reduction in the hepatic hematoma, with a right lobe volume of 614 cc and a left lobe volume of 158 cc (Figure 3). The patient continued regular follow-up in the Perinatology outpatient clinic.

Table 3 summarizes the clinical, biochemical, imaging, and therapeutic aspects of this case. It is important to note that preeclampsia was diagnosed late, as the patient already presented critical signs on Day 0. Despite this situation, and even in the presence of hypogastric pain, the cesarean section was performed in a facility that lacked the resources to manage a potential hepatic hematoma. Upon the patient’s transfer, the medical team faced critical decisions regarding whether a conservative or surgical approach would be more appropriate. On Day 3, the platelet count reached its lowest level, liver enzyme values remained elevated, and although blood pressure was controlled, the risk of hepatic bleeding persisted. Considering the patient’s hemodynamic stability, the multidisciplinary team chose a conservative strategy with hourly clinical evaluations. Biochemical monitoring showed partial improvement of HELLP syndrome by Day 10, which allowed the team to maintain this conservative approach. This decision was further supported by imaging evidence of hepatic hematoma resorption, as the volume began to decrease by Day 19.

**Table 3:** Timeline of important events ALT: alanine transaminase; AST: aspartate aminotransferase; D: day; S/D: systolic/diastolic

Group	Subgroup	Event (day)	D0	D2	D3	D6	D10	D11	D14	D18	D19	D20	D23	D27	D31	D37	D68
Clinical data	Symptoms	Abdominal pain	✔️	✔️	✔️	-	-	-	-	-	-	-	-	-	-	-	-
	Signs	Liver bleed	-	✔️	-	-	-	-	-	-	-	-	-	-	-	-	-
		Hypertension S/D mmHg	140/?	170/90	154/85	126/83	135/90	132/94									
		Hemodynamic stability	✔️	✔️	✔️	✔️	✔️	✔️	✔️	✔️	✔️	✔️	✔️	✔️	✔️	✔️	✔️
Biochemichal data	Liver enzyme elevation	ALT (UI/L)	-	296	311	90.8	67.1	32.1	32.6	20							
		AST (UI/L)	-	426	486	50.6	60.8	42.8	41.6	27.5							
		Platelet count (K/uL)	-	143	123	191	384	-	491	-	-	484	426	-	-	-	-
	Hemoglobin (g/dL)		-	11.9	9.4	8.4	9.9	-	9.8	-	-	9.3	9.9	-	-	-	-
Image data	Ultrasonography (right/left) cc		-	?/34	-	398/148	358/114	-	-	-	-	-	-	-	-	102/35	25/9
	Tomography (right/left) cc		-	-	1496/294	-	-	1550/131	-	-	1000/148	-	-	-	614/158	-	-

## Discussion

Although SHH is rare, since it has been reported in the context of preeclampsia or eclampsia, in other words, less than 1% of all pregnant women, it could lead to an extremely high mortality of more than 30% [9], associated with its rupture and lethal hepatic failure. Epigastric pain is one cardinal sign, and in 1844, this entity was named gastrodynia by Abercrombie [10,11]. In this case, this symptom was the red flag that augmented the clinical observation, even in the initial absence of hypertension.

SHH derives from a massive hepatic endothelial dysfunction. Hemolysis in the hepatic capillaries leads to platelet activation, forming a massive hematoma and microthrombi. The liver progressive necrosis cannot contain the hematoma; it ruptures, causing a massive intra-abdominal bleeding, and hypovolemic shock leads to maternal death. In this case, the hematoma reached 1800 cc in volume. However, biochemically, there were no signs of liver failure, suggesting no massive hepatic necrosis, and the Glysson capsule could contain the hematoma until it began to resolve spontaneously. 

**Figure 4:**
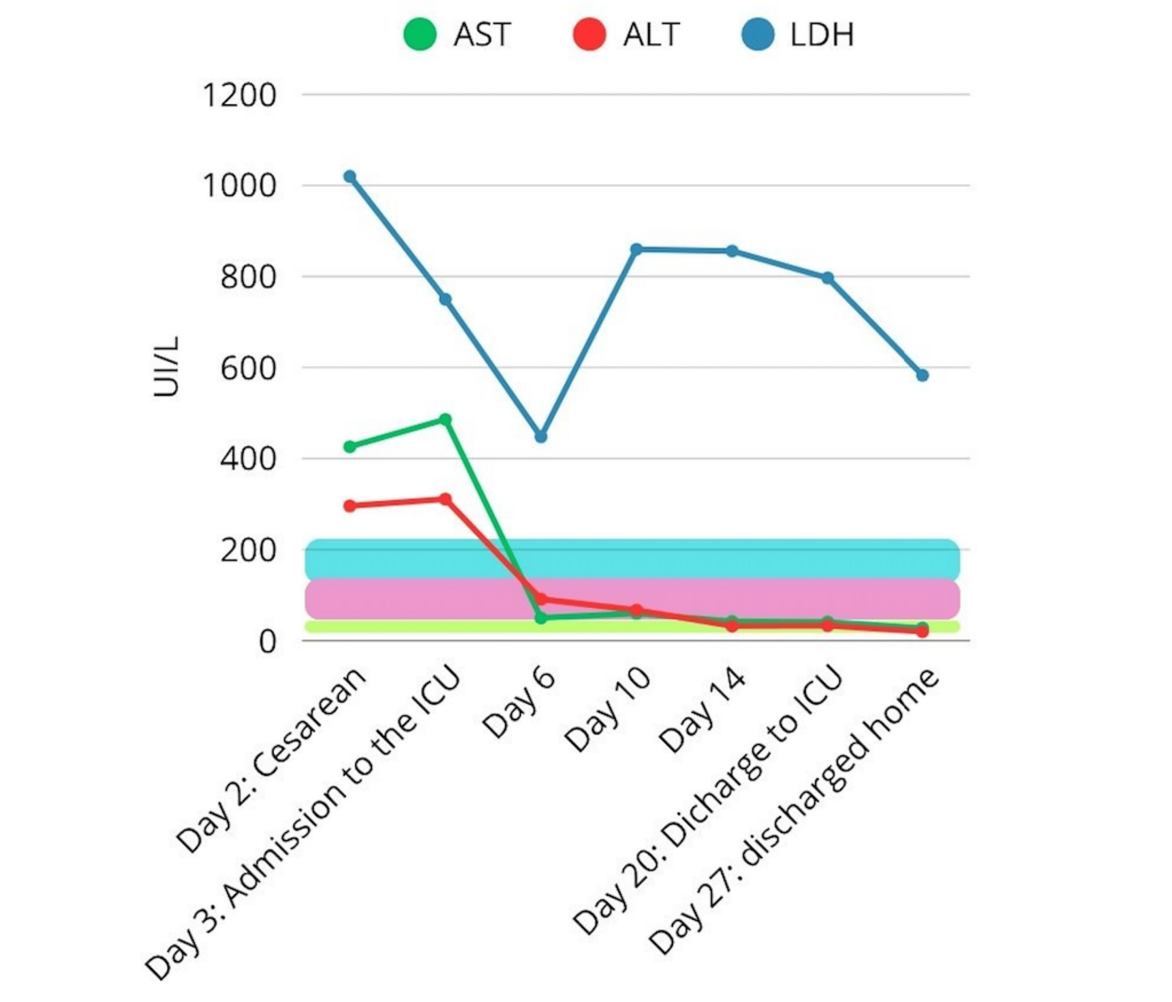
Serum liver enzyme trends (AST, ALT, LDH) during hospitalization, demonstrating progressive normalization following conservative management. Bands represent normal reference ranges. AST: aspartate aminotransferase; ALT: alkaline phosphatase; LDH: lactate dehydrogenase; ICU: intensive care unit

HELLP syndrome is composed of elevated liver enzymes (alanine transaminase (ALT), aspartate aminotransferase (AST), lactate dehydrogenase (LDH) and thrombocytopenia of less than 100,000 in the preeclampsia context [3]. In this case, the lowest platelet count was 123,000 (Figure 4), derived from platelet consumption by the hematoma. It could have been even lower, but the bone marrow of this patient balanced this consumption and allowed her to maintain her coagulation capacity. Although this value was above the 100,000 threshold typically required to establish a diagnosis of HELLP, the presence of other abnormal biochemical markers provided sufficient clinical evidence [9] to support the diagnosis of partial HELLP syndrome, which applies when not all diagnostic criteria are fully met.

A key indicator of potential hematoma rupture is a drop in hemoglobin levels [12]. In this case, the maximum drop was 3.5g/dL over four days. During the hospital observation, hemoglobin stabilized at 9.8 g/dL. She required a red blood cell concentrate with the lowest hemoglobin level of 8.4 g/dL; preeclampsia control and pregnancy resolution limited intravascular hepatic hemolysis.

The patient’s clinical course showed a clear correlation between biochemical parameters and the extent of the subcapsular hepatic hematoma. At presentation, transaminases and LDH levels were markedly elevated, and platelet counts were mildly reduced, coinciding with large hematomas in both hepatic lobes. Under conservative management and multidisciplinary follow-up, laboratory values steadily normalized, and the hematomas decreased substantially in size and volume, achieving partial resolution without surgical intervention.

Imaging assessment can be challenging in the context of an emergency like this. Ultrasound has become a readily accessible tool for identifying hepatic fluid collections and hematomas, playing a key role in the initial diagnosis and follow-up in this case, since one important treatment indication was the absolute rest. CT confirmed the hematoma diagnosis after cesarean section, since it is the gold standard for diagnosis of this condition.

The team performed hepatic arteriography to rule out active bleeding or arterial extravasation that would have required immediate radiologic or surgical intervention. In this case, the study confirmed the absence of active bleeding, which supported the decision to maintain conservative management, and it also provided detailed information on hepatic vascularization that guided clinical and imaging follow-up.

Management of SHH is complex, and treatment options should be selected based on the patient's clinical and hemodynamic stability, underlying etiology, extent of bleeding, hepatic function, coagulation status, and hemoglobin changes. In this case, the ICU team closely monitored blood pressure, heart rate, urine output, and clinical signs of peripheral hypoperfusion. The patient remained clinically stable, which justified the conservative management of the subcapsular hepatic hematoma, and no surgical approach was needed. 

Surgical treatment options include liver packing, embolization, or even a partial hepatectomy in severe cases [13]. In the event of rupture, which occurs in approximately one in 40,000 to one in 250,000 pregnancies, when hemodynamic instability is present, immediate surgical intervention is warranted due to the high associated maternal mortality [14]. If possible, a multidisciplinary team must be available at all times.

Because of the rarity of cases, each one is unique. This particular case showed that the hematoma was contained and there were no signs of active bleeding, like in other cases, where liver capsule rupture indicates mandatory surgery intervention to save the life [15]. In other cases, conservative methods have been successfully managed with excellent results, provided a complete unit and staff capacity is available [16]. Some case series have shown an 84% probability of success in conservatory management, making it possible for us to consider this treatment option [17].

Finally, in a review of 391 hepatic hematomas, it was observed that hemodynamic instability and eclampsia were related to maternal mortality; some patients did not have preeclampsia [18]. Conservative management with fatal outcome in cases like this may not be published due to editorial bias, since negative results tend to remain hidden from the literature. However, we suggest that every health professional attending cases like this should document their observations, as the rarity makes it difficult to gather sufficient evidence to decide in real clinical scenarios.

To this date, hepatic hematoma in pregnancy has no institutional protocol for management. In the scarcity of literature, the multidisciplinary team discusses each case to reach a consensus, and in every clinical or hemodynamic change, the team discusses again the best decision in order to protect the woman’s life.

We emphasize that conservative treatment in cases like this should be an option only if (i) there is a multidisciplinary team available at all times, (ii) there is an ICU capable of biochemical and clinical hemodynamic evaluation at least every hour, and (iii) the team has the capacity of rapid organization and team-derived decision. 

## Conclusions

Clinical high suspicion of SHH in pregnancy, even with mild preeclampsia or thrombocytopenia, can save lives. Ultrasound is a highly recommended and easy-to-perform imaging tool in cases like this. Finally, in selected patients with HELLP syndrome and SHH who are hemodynamically stable, have no active bleeding or capsular rupture on imaging, and show improving biochemical trends, conservative management can be safe and effective even with large or bilateral lesions. This case demonstrates that a structured, multidisciplinary approach-guided by close monitoring and predefined escalation criteria-can achieve recovery without surgical intervention. We suggest that surgical intervention must be reserved for cases with active bleeding, capsular rupture, or hemodynamic compromise.
